# The impact of scapular posture and sagittal spine alignment on motion and functional outcomes following reverse total shoulder arthroplasty: a scoping review

**DOI:** 10.1016/j.jseint.2024.02.009

**Published:** 2024-02-27

**Authors:** Bryan Sun, Justin Grad, Winnie Liu, Diane Nam, Ujash Sheth

**Affiliations:** a Michael G. DeGroote School of Medicine, McMaster University, Hamilton, ON, Canada; b Sunnybrook Orthopaedic Upper Limb (SOUL), Division of Orthopaedic Surgery, Department of Surgery, Sunnybrook Health Sciences Centre, University of Toronto, Toronto, ON, Canada

**Keywords:** Reverse total shoulder arthroplasty, Scapula, Spine, Posture, Shoulder, Arthroplasty, RTSA, Scapular posture

## Abstract

**Background:**

Reverse total shoulder arthroplasty (RTSA) has evolved beyond its initial indication for elderly patients with rotator cuff arthropathy and is now performed in younger patients for various shoulder pathologies. This surgical procedure has recently gained popularity and has been shown to result in similar functional improvements and complication rates compared to anatomical total shoulder arthroplasty. Scapular posture and sagittal spine alignment (SSPA) have recently emerged as factors potentially influencing RTSA outcomes. This scoping review aimed to assess the existing body of evidence on this topic.

**Methods:**

A systematic search was conducted on MEDLINE, Embase, and CENTRAL databases to evaluate the impact of scapular posture and SSPA on RTSA outcomes.

**Results:**

A total of 6 studies (616 shoulders) were included in this review. Scapular posture was found to influence RTSA outcomes, with studies reporting correlations between scapular posture with postoperative range of motion and functional scores. Suboptimal scapular posture, particularly type C (kyphotic posture with protracted scapulae), appeared to be associated with reduced external rotation. However, findings among the included studies regarding SSPA were varied. Some studies suggested that SSPA, notably thoracic kyphosis, might impact RTSA outcomes by influencing scapular posture, while others did not find a clear relationship.

**Conclusion:**

Scapular posture was implicated as a potential factor affecting RTSA outcomes; however, the role of SSPA remains inconclusive. There is currently a lack of high-quality evidence in the literature to draw definitive conclusions regarding the impact of scapular posture and SSPA on RTSA outcomes. More research is warranted to investigate these relationships more comprehensively.

Reverse total shoulder arthroplasty (RTSA) is a surgical procedure that was originally designed and indicated for treating elderly patients with rotator cuff arthropathy.^12^ RTSA is now being performed in younger patients for a wide variety of shoulder pathologies as a result of advancements in implant design and surgical expertise.^30^^,^^31^^,^^34^^,^^35^ Compared to anatomical total shoulder arthroplasty (ATSA), RTSA has been found to provide similar levels of functional improvement with similar complication rates.^9^ A recent study found that over 90% of patients are substantially satisfied with the results after RTSA.^26^ Consequently, the rates of RTSA surgery are increasing with the yearly number of procedures having nearly tripled between 2012 and 2017 in the United States.^3^

In RTSA, the conventional shoulder anatomy is altered by placing a glenosphere medially and an articulating concave humeral component laterally, effectively reversing the articulation of the shoulder.^8^ As a result, the center of rotation is medialized and inferiorized, resulting in an increased moment arm of the deltoid.^17^ As a result, the deltoid can function as the primary driver of shoulder movement and is able to compensate for deficiencies in the rotator cuff muscles.^22^ Significant postoperative recovery of flexion-extension range of motion (ROM), improved strength, and less pain is typically observed after surgery; however, rotational ROM often remains restricted.^16^ There are numerous preoperative factors that have been shown to correlate with postoperative success of the surgery. Postoperative American Shoulder and Elbow Surgeons (ASES) scores have been shown to be significantly higher in patients with the following baseline preoperative characteristics: higher ASES scores, less passive internal rotation, and greater active external rotation.^11^

A stable scapula is the thought to be the basis for glenohumeral motion as proper positioning of the scapula allows for optimal glenohumeral interaction.^21^ Dysfunctional positioning of the scapula, known as scapular dyskinesis, can lead to altered and disrupted shoulder function and stability.^25^ The alignment and mobility of the spine are closely connected to scapular positioning and can cause scapular dyskinesis.^14^^,^^36^ Thoracic spine posture in particular has been shown to affect scapular kinematics and can result in decreased muscle force.^15^ Subsequently, recent research has begun to explore the impact of scapular posture and sagittal spine alignment (SSPA) on RTSA outcomes. The purpose of this scoping review is to assess the current body of evidence relating to this topic.

## Materials and methods

A scoping review was conducted to evaluate the current understanding of the effect of scapular posture and SSPA on RTSA outcomes and identify gaps in knowledge to address in future studies. The PRISMA Extension for Scoping Reviews (PRISMA-ScR) checklist was used to ensure adherence to a validated methodology.^32^

### Search strategy

The search strategy was developed by one of the authors (BS) in collaboration with multiple health sciences research librarians. Given that scapular posture and SSPA are relatively new concepts in RTSA, the search strategy was kept intentionally broad. A single search of three databases (MEDLINE, Embase, and CENTRAL) from the earliest available date to present was conducted on April 21, 2023. (Supplementary Table S1)

### Study screening

All studies retrieved from databases were imported into Covidence (Veritas Health Innovation, Melbourne, Australia). Duplicate studies were automatically filtered out by the software. Three authors (BS, JG, and WL) reviewed all titles, abstracts, and full texts independently and in duplicate, with agreement being assessed at each stage. Discrepancies at each stage were resolved through consensus. If consensus was not reached, an expert in the field (US) was consulted for resolution. References of included articles were also assessed for inclusion eligibility.

### Inclusion/exclusion criteria

The inclusion criteria were (1) studies that measured scapular posture and/or SSPA, (2) studies reporting motion and/or functional outcomes following RTSA, (3) English studies, and (4) human, cadaveric, or computer-based studies. The exclusion criteria were (1) conference abstracts, (2) editorial articles, (3) reviews, and (4) commentaries.

### Data extraction

Data were independently abstracted by 3 authors (BS, JG, and WL) onto an Excel spreadsheet (Microsoft Corporation, Redmond, WA, USA). A random spot-check method was used for verification. Data extracted from each study included study characteristics (authors, title, year of publication, etc), patient demographics (age, sex), surgical information (implant type, indication), scapular posture and/or SSPA measurements, and outcome measures.

### Data synthesis

Inter-reviewer agreement at each stage was assessed using a kappa (κ) statistic, which was categorized as follows: 0 indicated no agreement, 0-0.2 indicated slight agreement, 0.2-0.4 indicated fair agreement, 0.4-0.6 indicated moderate agreement, 0.6-0.8 indicated substantial agreement, and 0.8-1.0 indicated almost perfect agreement.^37^ The primary aim of this study was to qualitatively assess the range of evidence relevant to our research question. We used descriptive statistics to summarize study characteristics and used a narrative approach to summarize relevant findings. As we were expecting a limited number of articles with heterogeneous outcome measures, we did not plan any meta-analyses.

## Results

### Selection of sources of evidence

**Figure 1:**
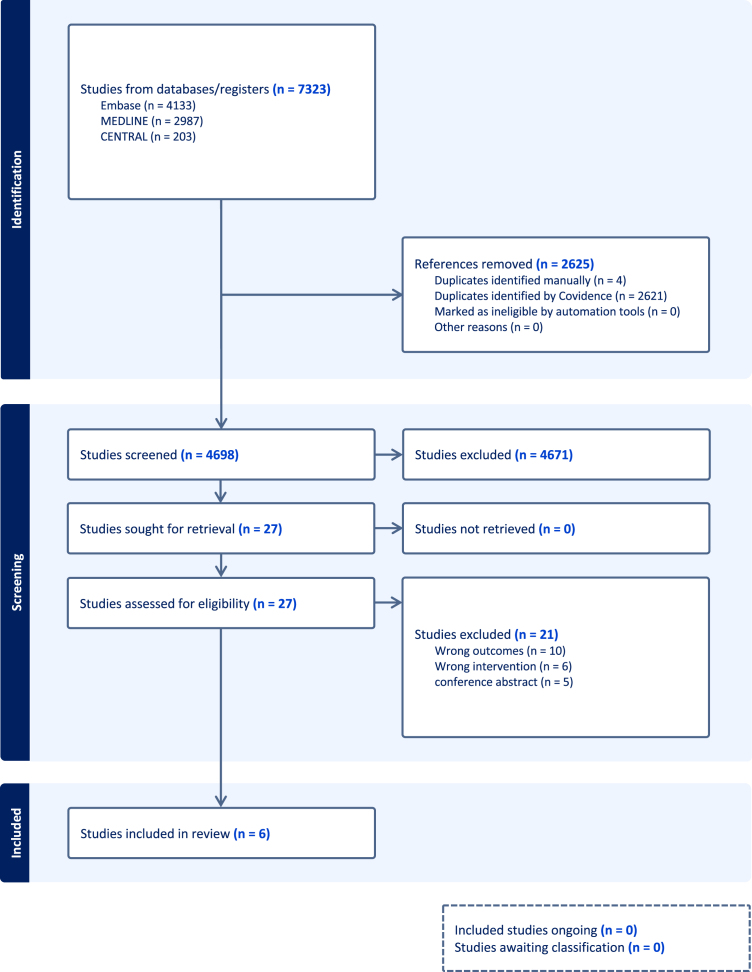
Preferred Reporting Items for Systematic Reviews and Meta-analyses (PRISMA) flow diagram representing a scoping review describing the impact of scapular posture and sagittal spine alignment on motion and functional outcomes following RTSA. *RTSA*, reverse total shoulder arthroplasty.

The systematic literature search of the online databases returned 4698 studies following the removal of duplicates. A total of 4671 studies were excluded during title and abstract screening leaving 27 full-text articles to be retrieved and screened for eligibility. Following this, 21 studies were excluded for the following reasons: 10 used the wrong outcomes, 6 used the wrong interventions, and 5 were conference abstracts. The remaining 6 studies were considered eligible for inclusion (Fig. 1). There was almost perfect agreement (κ = 0.92) at the title/abstract stage and perfect agreement (κ = 1.00) at the full-text stage.

### Characteristics of sources of evidence/patient demographics

The included articles comprised a total of 490 patients (616 shoulders) with females accounting for 261 shoulders (42.4%). The number of participants per study ranged from 14 to 279 and the number of shoulders per study ranged from 14 to 305. The mean participant age of each study ranged from 61.1 to 79.7 years. Of the included studies, 1 (16.7%) was a case–control study (Level III evidence), 2 (33.3%) were retrospective cohort studies (Level III evidence), and 3 (50%) were basic science studies. All included studies were published between the years 2020 and 2023. When stratifying by location, 2 studies (33.3%) were conducted in Germany, 2 (33.3%) were conducted in the United States, 1 (16.7%) was conducted in Japan, and 1 (16.7%) was conducted in Italy. The journals that the included studies were published in included *Journal of Shoulder and Elbow Surgery* (n = 3, 50%), *Clinical Orthopaedics and Related Research* (n = 1, 16.7%), *Journal of Orthopaedic Science: Official Journal of the Japanese Orthopaedic Association* (n = 1, 16.7%), *The Journal of the American Academy of Orthopaedic Surgeons* (n = 1, 16.7%). The characteristics of the 6 included studies are summarized (Table I).

**Table I:** Study characteristics.

Primary author	Publication year	Journal	Country	Level of evidence	# Of shoulders; patients	Mean age (y) +/− standard deviation	Sex (distribution)	Implants used
Moroder^19^	2020	Journal of Shoulder and Elbow Surgery	Germany	N/A	200 shoulders; 100 patients	61.1 (range, 18-89)	74 male; 26 female	N/A
Moroder^20^	2022	Clinical Orthopaedics and Related Research	Germany	N/A	30 shoulders	65 +/− 17	20 male; 10 female	Ascend Flex/Perform implant (Wright Medical Inc.)
Takayama^30^	2022	Journal of Orthopaedic Science	Japan	III	52 shoulders	80 +/− 5.09	22 male; 30 female	Trabecular Metal Reverse Shoulder System (Zimmer Biomet)
Reintgen^24^	2021	The Journal of the American Academy of Orthopaedic Surgeons	USA	III	305 shoulders; 279 patients	70.36 (range, 41-87)	149 male; 156 female	Equinoxe implant (Exactech)
Sulkar^29^	2023	Journal of Shoulder and Elbow Surgery	USA	N/A	15 shoulders	70.5 +/− 8.1	9 male; 6 female	Aequalis Reversed II (Strkyer, Kalamazoo), Delta Xtend Reverse Should System (Depuy-Synthes), and Zimmer Trabecular Metal Reverse Shoulder (Zimmer Biotmet)
Reina^23^	2023	Journal of Shoulder and Elbow Surgery	Italy	III	14 shoulders	N/A	7 male; 7 female	Aequalis Reverse Shoulder Prosthesis (Ascend flex, Stryker)

### Surgical information

Five studies provided information about the implants they used.^20^^,^^23^^,^^24^^,^^29^^,^^30^ These included the Ascend Flex/Perform implant (Wright Medical Inc., Memphis, TN, USA), the Trabecular Metal Reverse Shoulder System (Zimmer Biomet, Warsaw, IN, USA), the Equinoxe implant (Exactech, Gainsville, FL, USA), the Aequalis Reversed II (Stryker, Kalamazoo, MI, USA), and the Delta Xtend Reverse Shoulder System (DePuy-Synthes, Raynham, MA, USA). Additionally, 2 studies reported on the size of the glenosphere used. One clinical study reported a glenosphere diameter of 36 mm, while 1 computer simulation study used simulated glenosphere diameters of 36 mm and 42 mm.^20^^,^^30^

### Scapular orientation

Moroder et al (2020) created 3-dimensional models of 200 shoulders with whole-body computed tomography (CT) scans. The relevant scapular posture measurements were scapular protraction, scapular internal rotation, scapular upward rotation, scapular translation, scapular tilt, humeral torsion, and glenoid version. The outcome was the correction angle where a virtually implanted humeral component would be in neutral opposition to the arm in neutral rotation. Significant correlations were found between the correction angle and scapular internal rotation (R = 0.71 *P* < .001), protraction (R = 0.39, *P* < .001), upward rotation (R = 0.16, *P* = .03), translation (R = −0.14, *P* = .04), and tilt (R = 0.22, *P* = .02). The authors also proposed a postural classification system based on the Gaussian distribution of scapular internal rotation. Type B (intermediate posture) was defined as mean scapular internal rotation ± 1 standard deviation (36.2°-46.6° of internal rotation), type A (upright posture with retracted scapulae) was the lower bound (<36.2° of internal rotation), and type C (kyphotic posture with protracted scapulae) was the upper bound (>46.6° of internal rotation).^19^

Moroder et al (2022) similarly used whole-torso CT scans of 30 patients to generate virtual models which were stratified by scapular posture. A short, curved, metaphyseal fitting stem was then virtually implanted into each shoulder model and varying humeral component retrotorsion angles, neck-shaft angles, and glenosphere types were used to simulate 3720 different RTSA configurations. A total of 660 were classified as patients with type A posture (n = 5), 1650 to patients with type B posture (n = 13), and 1410 to patients with type C posture (n = 12). The outcome measures were ROM in adduction, abduction, flexion, extension, internal rotation, external rotation, hand to the contralateral side, combing hair, and hand to the back pocket motions. A combined motion score (sum of median degrees of motion for all planes examined) was also calculated. The authors reported that regardless of RTSA implant configuration, posture type had a strong effect on ROM in all planes except for flexion. Of particular interest, when compared to type A, type C posture had inferior adduction (median 5° [interquartile range (IQR) −7 to 20°] vs. 15° [IQR 7°-20°]; *P* < .01), abduction (63° [IQR 48°-78°] vs. 72° [IQR 63°-82°]; *P* < .01), extension (4° [IQR −8° to 12°] vs. 19° [IQR 8°-27°]; *P* < .01), and external rotation (7° [IQR −5° to 22°] vs. 28° [IQR 13°-39°]; *P* < .01), but superior internal rotation (104° [IQR 92°-121°] vs. 92° [IQR 80°-102°]; *P* < .01) and combing hair motion (36° [IQR 27°-36°] vs. 27° [IQR 27°-36°]; *P* < .01).^20^

Reina et al (2023) sought to study how scapular motion contributes to functional outcomes after RTSA. Fourteen patients who underwent RTSA were included and were stratified by the posture classification system devised by Moroder et al (2020).^19^ Eight patients were type B, 6 were type C, and 0 were type A. The authors measured each patient’s postoperative ROM in flexion, abduction, and internal/external rotation while simultaneously recording scapular motion in 3 planes (protraction/retraction, anterior/posterior tilt, and upward/downward rotation). Average postoperative flexion was 152.3 degrees, abduction was 143.5 degrees, and external rotation was 25 degrees. Internal rotation was measured by the spinal level the thumb could reach behind the back: 12 patients were T12, 2 were L1, and 2 were gluteus. In terms of scapular motion, RTSA shoulders demonstrated more prominent posterior tilting, anticipation of scapular retraction, and greater upward rotation in both flexion and abduction. Therefore, changes in scapulothoracic posture and motion may be required to optimize post-RTSA functional outcomes as they help compensate for reduced glenohumeral motion.^23^

Sulkar et al (2023) measured degrees of scapulothoracic upward rotation, protraction, and tilt, along with various humerothoracic and glenohumeral joint orienftations following RTSA. Patients were separated into 2 groups based on their shoulder’s internal rotation capability. The high group consisted of patients who were able to perform internal rotation in adduction with their hand behind their back to spinal level T12 or higher, and the low group consisted of the patients who could not. All patients were then imaged with biplane fluoroscopy with the shoulder in both neutral and internally rotated positions. Key findings were that in neutral position, the high group had on average 9 degrees less posterior tilt (*P* = .017) and 7 degrees more upward rotation (*P* = .100). The high group also had significantly greater glenohumeral elevation in internal rotation (*P* = .047) in neutral pose, a greater plane of elevation (*P* = .002) and axial rotation (*P* = .001) in internal rotation pose. Mean postoperative Simple Shoulder Test (SST) score was 11 ± 1 in the high group and 9 ± 2 in the low group (*P* = .019). Mean postoperative ASES score was 94 ± 13 in the high group and 83 ± 16 in the low group (*P* = .178).^29^

### Sagittal spine

Moroder et al (2020) also assessed thoracic kyphosis as a predictor of ROM following RTSA. The authors found a significant correlation between thoracic kyphosis and the correction angle required for neutral opposition of the humeral component relative to the glenosphere (R = 0.14, *P* = .04) as well as with scapular internal rotation (R = 0.27, *P* < .001). Additionally, after categorizing patients by posture type (A, B, and C), type C patients were found to have significantly greater thoracic kyphosis than type A (*P* = .001) and type B (*P* = .02).^19^

Moroder et al (2022) validated their previous findings by once again identifying significant differences in thoracic kyphosis across the 3 scapular posture categories (type A = 36 ± 7°, type B = 45 ± 13°, type C = 44 ± 14°, *P* = .02). Posture type was found to strongly affect all ROM planes except flexion. In particular, type C patients had reduced adduction, abduction, extension, and external rotation; however, they had improved internal rotation and combing hair motion compared to type A patients.^20^

Reintgen et al (2021) measured RTSA patients’ preoperative degrees of thoracic kyphosis and stratified them into 3 groups (kyphosis <25°, kyphosis 25°-45°, and kyphosis >45°). Preoperative and final follow-up ROM (forward elevation, abduction, internal rotation, external rotation), patient-reported outcome measures (SPADI, SST012, ASES, UCLA, SF012, visual analog scale), and the Constant score were collected. The authors reported no statistically significant differences in preoperative or postoperative delta ROM measurements, patient-reported outcome measures, or Constant score between the 3 groups.^24^

Takayama et al (2022) evaluated SSPA based on 10 parameters. These were thoracic kyphosis, lumbar lordosis, thoracic-lumbar angle ratio, pelvic incidence, pelvic tilt, C7-HA (line from C7 plumb line to hip axis), C7-SVA (line from C7 plumb line to posterosuperior corner of the sacrum), T1-STA (angle between T1 plumb line and line connecting T1 to hip axis), T9-STA (angle between T9 plumb line and line connecting T9 to hip axis), and spinal tilt. ROM measurements (flexion, abduction, internal rotation, external rotation) were collected preoperatively and at the last follow-up. Patients were then categorized into 2 groups: 1 where internal rotation deteriorated after RTSA (group A) and 1 where it did not (group B). Between the 2 groups, statistically significant differences were found for lumbar lordosis, thoracic-lumbar angle ratio, C7-HA, C7-SVA, T1-STA, and spinal tilt. There were no between-group differences in preoperative ROM, but group A had significantly reduced ROM in all movements except for external rotation. Linear multiple regression analysis was conducted and found no explanatory variable that was strongly correlated with postoperative internal rotation ROM. Logistic regression analysis demonstrated that C7-HA was correlated with internal rotation deterioration (odds ratio = 1.95, 95% confidence interval 1.33-2,84, *P* < .001). A receiver operating characteristic curve was used to identify a C7-HA cutoff value of 2.44 cm (sensitivity = 91.3%, specificity = 93.1%, *P* < .001, area under the curve = 0.938).^30^

## Discussion

This scoping review represents a mapping of the current body of evidence regarding the impact of scapular posture or SSPA on motion and functional outcomes after RTSA. Our 2 main themes, scapular posture and SSPA, are relatively new concepts in relation to RTSA optimization.^2^ Thus, our primary aim was to descriptively highlight early trends and findings, and suggest directions for future research.

Four studies reported on scapular posture and its impact on motion or functional outcomes after RTSA.^19^^,^^20^^,^^23^^,^^29^ While different authors operationalized scapular posture differently and used unique outcome measures, the general trend was that scapular posture did have an impact on post-RTSA outcomes. The first group to assess this relationship conducted 2 CT-based computer modeling studies. In the first, they found that scapular internal rotation had a large positive correlation with the correction angle required to achieve neutral opposition of the humeral component following RTSA.^19^ Logically, achieving neutral opposition should maximize postoperative internal and external rotation by reducing impingement in either direction. Thus, if patients have differing scapular postures, using the same RTSA techniques and configurations would not consistently achieve neutral opposition, leading to deficits in postoperative ROM. On this basis, the authors proposed a classification system of 3 scapular posture types based on scapular internal rotation. This system was then tested in their second study where patients were stratified by posture type before virtually simulated RTSA. The authors reported that simulated ROM was strongly affected by posture type in all planes of motion except for flexion. Interestingly, they also reported that despite having inferior external rotation, type C posture had superior internal rotation and combing hair ROM compared to type A posture.^20^ This seemingly implies that internal rotation drives the hair combing motion, whereas in reality, external rotation is actually more important.^33^ This finding may be attributed to the fact that hair combing ROM was compared using medians instead of means. The reported IQRs for hair combing ROM is identical between the 2 groups, potentially indicating that the reported difference is inaccurate. In any case, further research is warranted to clarify this finding. Another study also categorized RTSA patients in the same way with all either having type B or type C posture. Although the outcomes were not stratified by posture type, overall postoperative ROM averages were reported. In particular, mean external rotation was 25 degrees, which is lower than the optimal 30 degrees as reported in the literature.^4^^,^^23^ Previous studies have demonstrated significantly inferior external rotation in type C posture compared to type A. Thus, the external rotation deficit seen in this study could be attributed to the increased protraction of type B and type C scapulae. This study also reported greater scapular tilt and retraction starting at 30° of flexion and abduction in RTSA shoulders. This postoperative adaptation may help generate a more favorable moment arm for the deltoid to generate glenohumeral motion. As such, these findings suggest that striving to recreate native scapular posture or kinematics during RTSA may be fundamentally flawed. A final study dichotomized RTSA patients based on postoperative internal rotation (high vs. low). Significant between-group differences in postoperative scapulothoracic orientations were found, highlighting a link between scapular posture and post-RTSA internal rotation. From a functional perspective, the mean SST score was significantly different between groups.^29^ However, the minimally clinical important difference for the SST score has been reported in studies to range from 2.4 to 3.^13^^,^^31^ Thus, while statistically significant, the between-group difference in mean SST may not be clinically significant.

Four studies reported on SSPA and its impact on motion or functional outcomes after RTSA.^19^^,^^20^^,^^23^^,^^30^ Interestingly, the included studies had conflicting results regarding whether SSPA impacts RTSA outcomes. One study found a weak correlation between thoracic kyphosis and the correction angle required for neutral opposition post-RTSA (R = 0.14) and a slightly stronger correlation between thoracic kyphosis and scapular internal rotation (R = 0.27). Thoracic kyphosis was also shown to significantly differ across scapular posture types.^19^ In a follow-up study, scapular posture was again shown to correlate with thoracic kyphosis and was also shown to strongly affect simulated post-RTSA ROM.^20^ While these 2 studies did not provide evidence that thoracic kyphosis directly influences RTSA outcomes as an independent predictor, they did show that thoracic kyphosis influences scapular posture, which is in line with the literature.^14^^,^^28^^,^^36^ Another study found no significant differences in postoperative ROM, patient-reported outcome measures, or Constant score between groups that were stratified by preoperative thoracic kyphosis.^23^ This was in stark contrast to the authors’ predictions as it has been established that RTSA leads to diminished overhead ROM in the native shoulder. A final study used multiple regression analysis to show that SSPA was not strongly correlated with postoperative internal rotation. However, logistic regression analysis did show that C7-HA was correlated with internal rotation deterioration.^30^ Previous studies have shown that SSPA deformations reduce the strength of periscapular muscles such as the trapezius, rhomboids, and latissimus dorsi which facilitate shoulder internal rotation.^1^^,^^27^ SSPA has also been shown to influence native shoulder ROM directly via its anatomical articulations.^6^ It is noteworthy that, to the best of our knowledge, there is no existing literature examining the influence of scapular and spinal posture on outcomes in ATSA. We hypothesize that posture would play a relatively minor role in ATSA outcomes compared to reverse RTSA, as ATSA is inherently less constrained based on the design.^10^^,^^18^ Nevertheless, further research is warranted to substantiate this hypothesis and facilitate a comparative analysis with RTSA.” This study aimed to explore this relationship, but its findings neither proved nor disproved its validity. As such, further research is required to reach a definitive conclusion.

The limitations of this study mainly arose from the limited number and quality of included studies. Only 6 studies were included in this review and none were prospective or randomized in nature. An essential limitation to recognize is the variation in implant types across the included studies. Different implant designs can lead to diverse postoperative biomechanical forces.^5^ Consequently, the impact of scapular and sagittal spine posture on functional outcomes may vary based on the unique characteristics of each implant. Moreover, the studies included in the analysis did not provide details about the mechanisms employed to validate the accuracy of implant placement. This is a crucial aspect, as the precision of implant positioning is known to significantly influence postoperative outcomes. Discussing this limitation is particularly pertinent, given that obtaining accurate implant positioning is typically challenging, and we posit that it might be even more challenging in individuals with abnormal postures.^7^ Additionally, no quantitative analysis was performed as the data could only be presented descriptively given the heterogeneity in outcome measures. Lastly, risk of bias was not assessed as the majority of studies were basic science studies. This meant that this scoping review could not report on the methodological quality of the current literature. Future research should focus on further elucidating the relationships between scapular posture, SSPA, and functional and motion outcomes following RTSA using well-designed prospective or retrospective studies. Special consideration should be taken to standardize evaluation criteria to properly enable comparisons and systematic data syntheses. The role of the implant positioning across different postures, implant type (eg, in-lay vs. on-lay), and comparisons with ATSA should also be addressed in future studies.

## Conclusion

This scoping review demonstrated that there is a paucity of research on the impact of scapular posture and SSPA on motion and functional outcomes after RTSA. While included studies did report findings that suggest a possible relationship between scapular posture and SSPA with RTSA outcomes, there currently is insufficient high-quality evidence to draw strong conclusions from. As such, this review recommends the consideration of well-designed prospective or retrospective studies to further investigate this topic.

## Disclaimers:

Funding: No funding was disclosed by the authors.

Conflicts of interest: The authors, their immediate families, and any research foundation with which they are affiliated have not received any financial payments or other benefits from any commercial entity related to the subject of this article.
