# Supplementary material for: The impact of scapular posture and sagittal spine alignment on motion and functional outcomes following reverse total shoulder arthroplasty: a scoping review
Source: JSES Int. 2024 Feb 27;8(4):859–65. doi: 10.1016/j.jseint.2024.02.009 (PMC11258846; doi:10.1016/j.jseint.2024.02.009)
Supplement: Supplementary Table S1 [file mmc1.docx]

| **MEDLINE (n=2897)** | **Embase (n=4133)** | **CENTRAL (n=203)** |
| --- | --- | --- |
| 1. exp “Range of Motion, Articular”/ | 1. “range of motion”/ | 1. exp “Range of Motion, Articular”/ |
| 2. motion*.ti,ab,kf. | 2. motion*.ti,ab,kf. | 2. motion*.ti,ab,kf. |
| 3. function*.ti,ab,kf. | 3. function*.ti,ab,kf. | 3. function*.ti,ab,kf. |
| 4. postural mechanic*.ti,ab,kf. | 4. postural mechanic*.ti,ab,kf. | 4. postural mechanic*.ti,ab,kf. |
| 5. shoulder kinematic*.ti,ab,kf. | 5. shoulder kinematic*.ti,ab,kf. | 5. shoulder kinematic*.ti,ab,kf. |
| 6. Biomechanical Phenomena/ | 6. biomechanics/ | 6. Biomechanical Phenomena/ |
| 7. (shoulder adj2 (arthroplasty* OR replace*)).ti,ab,kf. | 7. (shoulder adj2 (arthroplasty* OR replace*)).ti,ab,kf. | 7. (shoulder adj2 (arthroplasty* OR replace*)).ti,ab,kf. |
| 8. Arthroplasty, Replacement, Shoulder/ | 8. total shoulder arthroplasty/ OR reverse shoulder arthroplasty/ OR shoulder arthroplasty/ | 8. Arthroplasty, Replacement, Shoulder/ |
| 9. 1 OR 2 OR 3 OR 4 OR 5 OR 6 | 9. 1 OR 2 OR 3 OR 4 OR 5 OR 6 | 9. 1 OR 2 OR 3 OR 4 OR 5 OR 6 |
| 10. 7 OR 8 | 10. 7 OR 8 | 10. 7 OR 8 |
| 11. 9 AND 10 | 11. 9 AND 10 | 11. 9 AND 10 |
